# TMEM16A: An Alternative Approach to Restoring Airway Anion Secretion in Cystic Fibrosis?

**DOI:** 10.3390/ijms21072386

**Published:** 2020-03-30

**Authors:** Henry Danahay, Martin Gosling

**Affiliations:** Enterprise Therapeutics, Science Park Square, Brighton BN1 9SB, UK; martin@enterprisetherapeutics.com

**Keywords:** calcium-activated chloride channel, CFTR, mucus clearance, ion transport, TMEM16A potentiator, ETX001

## Abstract

The concept that increasing airway hydration leads to improvements in mucus clearance and lung function in cystic fibrosis has been clinically validated with osmotic agents such as hypertonic saline and more convincingly with cystic fibrosis transmembrane conductance regulator (CFTR) repair therapies. Although rapidly becoming the standard of care in cystic fibrosis (CF), current CFTR modulators do not treat all patients nor do they restore the rate of decline in lung function to normal levels. As such, novel approaches are still required to ensure all with CF have effective therapies. Although CFTR plays a fundamental role in the regulation of fluid secretion across the airway mucosa, there are other ion channels and transporters that represent viable targets for future therapeutics. In this review article we will summarise the current progress with CFTR-independent approaches to restoring mucosal hydration, including epithelial sodium channel (ENaC) blockade and modulators of SLC26A9. A particular emphasis is given to modulation of the airway epithelial calcium-activated chloride channel (CaCC), TMEM16A, as there is controversy regarding whether it should be positively or negatively modulated. This is discussed in light of a recent report describing for the first time *bona fide* TMEM16A potentiators and their positive effects upon epithelial fluid secretion and mucus clearance.

## 1. Introduction

Cystic fibrosis (CF) is a recessive genetic multiorgan disease that is primarily associated with pulmonary, gastrointestinal and reproductive tract dysfunction [1]. CF is estimated to affect >75,000 patients globally and is caused by loss-of-function mutations in the anion channel, cystic fibrosis transmembrane conductance regulator (CFTR). The present model of airway disease in CF is based on the concept that inadequate CFTR function results in insufficient hydration of the airway mucosa [2]. In this desiccated environment, the mucus gel has a higher percentage solid and tends to adhere to the epithelium, resulting in impaired mucus clearance by both mucociliary and cough action [3]. Under these conditions, there is an increased risk of infection and colonization of the airways by inhaled pathogens. Furthermore, stagnant mucus can lead to direct plugging and gas trapping in the smaller airways. The concept that inadequate hydration of the airway mucosa drives CF lung disease throughout life is likely an oversimplification, as additional factors such as loss of alkalinisation of the mucosa through impaired CFTR-mediated bicarbonate secretion may also contribute to the disease phenotype. An acidic pH in the airway has been demonstrated to both reduce bacterial killing by pH-sensitive host factors and also to prevent the unfolding of mucin macromolecules upon secretion [4,5,6]. However, the relevance of airway pH is controversial, as recent work has demonstrated no differences between children with or without CF [7].

Clinical studies with inhaled hypertonic saline, a therapy that will promote mucosal hydration, support the concept that increasing available fluid in the airway will improve mucus clearance and lung function and reduce exacerbation frequency [8,9]. More recently, the concept that restoring airway epithelial anion secretion through the repair of CFTR has been clinically validated with both CFTR potentiator and corrector molecules [10]. Perhaps the most compelling and complete data to date have been established with the CFTR potentiator molecule ivacaftor (VX-770) in patients heterozygous for a gating mutation in CFTR (e.g., G551D and R117H). In these patients, significant improvements in airway epithelial anion transport in vitro [11] have translated through to clinical improvements in mucus clearance [12], reduced mucus plugging [13], increases in lung function and a reduced rate of disease exacerbations [14,15]. Presently, CFTR repair therapies offer the potential to treat up to 90% of the CF population. However, minimally 10% of CF patients have genotypes that will remain refractory to these therapies and represent a significant unmet medical need. Furthermore, for the 90% of the CF patient population who are genetically suited to existing CFTR repair drugs, their disease is not cured, and significant unmet medical need remains [16]. To this end, there is a powerful argument to look for alternative, non-CFTR-based therapies that would be agnostic to any given CFTR mutation and therefore suitable for all CF patients [17].

## 2. CFTR-Independent Approaches to Restoring Airway Mucosal Hydration

CFTR undoubtedly plays a salient role in the regulation of anion and thereby fluid secretion across the airway mucosa. However, several additional ion channels and transporters are involved in the maintenance of mucosal hydration and may therefore represent targets for future therapies. That mucus clearance is impaired but not completely inhibited in the CF lungs [18] supports the concept that additional hydration mechanisms do exist even in the absence of normal CFTR function.

The epithelial sodium channel (ENaC) has been the target of many drug discovery efforts to promote mucus clearance [19]. ENaC is responsible for sodium and fluid absorption out of the airway. Inhibiting ENaC function is therefore predicted to reduce dehydration and promote mucus clearance. In support of the target validation, patients with loss of function mutations in ENaC subunits have a salt-wasting disease, pseudohypoaldosteronism type 1, but also exhibit accelerated rates of airway mucociliary clearance [20]. Inhaled dosing with amiloride, a potassium-sparing diuretic and ENaC blocker, has been demonstrated to accelerate mucociliary clearance in clinical studies [21]. Conversely, airway-specific overexpression of βENaC to phenocopy the increased sodium transport characteristic of CF airways resulted in CF-like lung disease in mice [22,23]. Despite what appears to be a strong target validation for ENaC, a host of negative regulators of the channel, including amiloride, VX-371, QBW276 and SPX-101, have failed to show robust clinical benefit. The lack of clinical benefit with inhaled amiloride, the first ENaC blocker to be tested in CF, was ascribed to a short duration of action in the airways [24]. Increasing the dose or dosing frequency of amiloride were not viable approaches to extend the duration of action in the airway, as ENaC is also expressed in the cortical collecting duct of the kidney where it regulates sodium and potassium exchange [25]. Blocking ENaC in the kidney can result in a potentially life-threatening accumulation of potassium (hyperkalaemia). Subsequent generations of inhaled ENaC blockers have been designed to either be retained in the lung and/or to be cleared from the systemic circulation by nonrenal mechanisms. Unfortunately, clinical studies with these post-amiloride candidates have not included endpoints to confirm whether the selected doses have effectively blocked ENaC in the airways, making interpretation of the negative clinical result problematic. It remains an open question as to whether airway ENaC is a flawed target for the treatment of CF lung disease or whether the compounds tested to date have been inadequate. One hypothesis to explain the lack of clinical benefit with the inhaled ENaC blockers tested to date is simply that they have been underdosed. An analysis of the efficacious doses of the likely clinical candidates in a sheep model of mucociliary clearance suggests just that [26]. Moving forward with future candidates, it would perhaps be wise to include an assessment of target engagement in the lung in an acute clinical setting using an endpoint such as mucociliary clearance before commencing longer-term studies with lung function endpoints.

SLC26A9, a member of the solute carrier 26 family, may also contribute to anion and fluid secretion in the airway epithelium [27,28]. SLC26A9 has been demonstrated to transport chloride ions through both CFTR-dependent and -independent mechanisms, although a lack of specific pharmacological tools has limited our understanding of its function. Genetic evidence supports SLC26A9 as a disease modifier in CF, and model systems have demonstrated a significant role of this anion channel in the regulation of mucus hydration [29]. Upregulation of *Slc26a9* expression in airway inflammation protects mice from airway mucus plugging whilst knockout animals show a severe plugging phenotype [30]. Positive and negative regulators of SLC26A9 function are required to validate the transporter as a drug target to treat mucus obstruction and to understand its therapeutic potential.

The existence of a calcium-activated chloride conductance in the human airway epithelium has been recognised for almost 30 years. Agents that would promote anion secretion through the CaCC may provide sufficient drive to hydrate the airway mucosa in the absence of CFTR. Historically, a number of potential molecular candidates encoding the epithelial CaCC have been proposed, including CLCA1, ClC3 and bestrophin 1. CLCA1 is now accepted to be a secreted protein rather than an ion channel [31], whilst ClC3 is expressed intracellularly and functions as a Cl/H exchanger [32]. Bestrophin 1 has a biophysical fingerprint that is different to the native CaCC, ruling it out as a candidate for the native airway CaCC [33]. In 2008, TMEM16A was proposed as a CaCC with a biophysical fingerprint and expression pattern that was consistent with the airway native CaCC [34,35,36]. Of note, CLCA1 has been reported to bind and stabilise TMEM16A and so may play an indirect regulatory role in calcium-activated anion secretion [37]. In support of TMEM16A as the identity of the CaCC, gene silencing attenuated CaCC activity in cultured cells and tissues including airway epithelia and salivary glands [34,36]. *Tmem16a* knockout mice also showed attenuated CaCC-mediated function in isolated tracheas when stimulated with purinergic agonists [38]. Most recently, a TMEM16A potentiator compound, ETX001, has been demonstrated to enhance the calcium-dependent chloride current in cells expressing recombinant TMEM16A and in primary CF bronchial epithelial cells [39]. Of note, a tight correlation was observed between the recombinant TMEM16A system and the native CaCC assay in CF cells, which further supports the identity of the native human airway epithelial CaCC as being TMEM16A.

**Figure 1:**
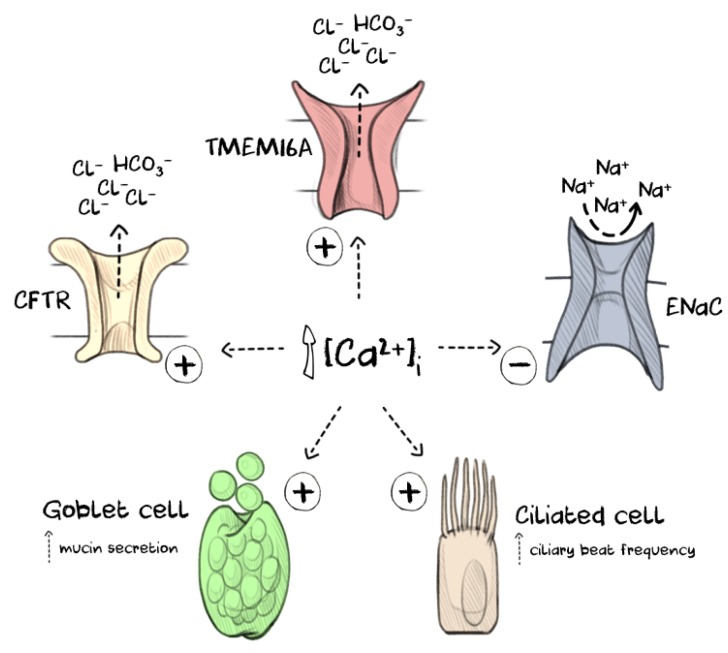
Increasing intracellular calcium levels [Ca^2+^]_i_ in the airway epithelium impacts several processes that regulate mucus clearance. Intracellular calcium signalling drives goblet cell exocytosis to release gel-forming mucins (commonly MUC5AC and MUC5B) onto the airway mucosa where they become rapidly hydrated and form a mucus gel. The fluid for hydration is provided by the coordinated activity of several ion channels, including cystic fibrosis transmembrane conductance regulator (CFTR), epithelial sodium channel (ENaC) and TMEM16A. Elevated intracellular calcium levels activate anion secretion through both TMEM16A and CFTR and attenuate sodium reabsorption by inducing internalisation of ENaC. The mucus gel is then cleared along the airways by the coordinated beating of cilia on the multiciliated cells, the frequency of which is also increased through calcium signalling.

Historically, TMEM16A has been indirectly targeted by drug candidates designed to enhance mucus clearance in the CF airway. Denufosol (INS-37217), a P2Y2 receptor agonist, and duramycin (Moli-1901), a calcium ionophore, elevate intracellular calcium levels when applied to airway epithelial cells, which has a number of effects to promote mucus clearance (Figure 1), including the opening of TMEM16A [40,41], activation of CFTR-mediated secretion [42], inhibition of ENaC function [43], goblet cell exocytosis and mucin secretion [40,44] and an increase in ciliary beat frequency [45]. Ultimately, denufosol failed to show robust benefit in the CF airway, and development was stopped [46], whilst there have been no reports of the continued development of Moli-1901 for several years [47]. In the case of denufosol, the P2Y2 receptor was found to rapidly desensitise, and intracellular calcium stores were depleted following dosing, limiting the duration of the opening of TMEM16A [48,49]. Furthermore, stimulation of mucin release from airway goblet cells may have negatively impacted the benefit of any enhanced hydration provided by denufosol [40]. It has also been recently suggested that some of the benefit derived from inhaled hypertonic saline may be TMEM16A mediated [50]. Using a CF pig model (CFTR^−/−^), approximately 50% of the fluid accumulation in the airways was mediated by the stimulation of airway neurons leading to epithelial secretion rather than through a direct osmotic effect. Based on these findings, a drug that would potentiate the opening of TMEM16A but without raising intracellular calcium levels would be predicted to further enhance anion secretion and could be used to examine the therapeutic hypothesis that the airway CaCC could be utilised to bypass CFTR in CF lung disease. However, TMEM16A function is unlikely to be restricted to anion secretion in the airway epithelium. A role for TMEM16A has been proposed in the mechanics of goblet cell exocytosis [51,52], in the stimulation of goblet cell formation [53,54,55] and in the function of smooth muscle and neuronal tissues [56]. In the remainder of this review, we will focus in more detail on TMEM16A as a potential drug target, describe the profile of a recently described TMEM16A potentiator molecule, ETX001, and discuss some of the controversy surrounding the biological functions of TMEM16A beyond anion secretion.

## 3. TMEM16A: Regulation of CaCC Function in the Airway Epithelium

**Figure 2:**
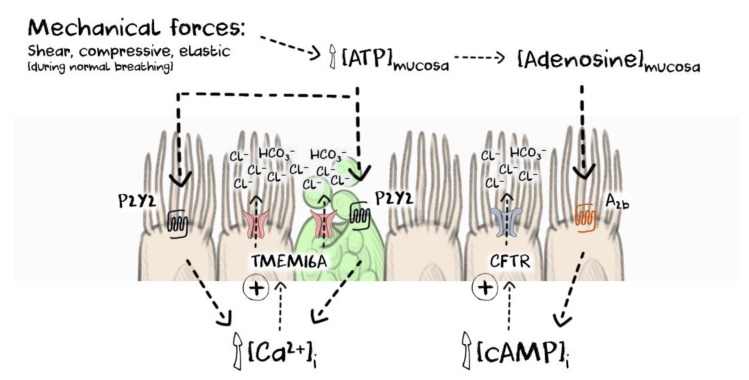
The current model describing the local regulation of calcium-dependent signalling in the human airway epithelium. Endogenous ATP is released from epithelial cells in the airways during normal breathing and is regulated by compressive, elastic and shear forces. ATP binds to P2Y2-receptors and elevates local [Ca^2+^]_i_ leading to activation of TMEM16A and CFTR to promote anion and fluid secretion and also enabling goblet cell exocytosis to provide mucins to form the mucus gel. ENaC function is also attenuated when intracellular calcium is elevated (not shown for clarity). Mucosal ATP is also metabolised to adenosine and can further activate CFTR through an adenosine A2b-cAMP mediated pathway. As the rate and depth of breathing increase, ATP levels rise to promote fluid secretion to compensate for evaporative loss and to provide an increased quantity of a well-hydrated mucus gel to clear the increased burden of inhaled particles. [cAMP]_i_ = intracellular cAMP.

The first detailed characterisation of a CaCC in a secretory epithelium [57] described a conductance that was both voltage- and calcium-dependent and was proposed as the major chloride conductive pathway in the apical membrane of rat lacrimal glands following muscarinic stimulation. In the human airway, calcium ionophores and purinergic agonists were demonstrated to likewise stimulate a calcium-dependent secretory current in both normal and CF derived cells [58,59,60] and also to elicit a hyperpolarisation of the nasal potential difference (NPD) when perfused over the nasal mucosa of CF patients [61]. The endogenous production and release of purinergic ligands, particularly ATP and UTP, in the airway mucosa appears to be central to the regulation TMEM16A function [48,62,63]. Mechanical forces established during normal breathing (compressive, elastic and shear) have been demonstrated to increase levels of purinergic ligands in the airway-lining fluid and in turn regulate ion transport processes and promote fluid secretion (Figure 2). As described above, these effects are not exclusively TMEM16A-mediated, as elevated intracellular calcium will also influence CFTR and ENaC function (Figure 1), and the metabolism of ATP to adenosine in the airway mucosa will activate CFTR through an adenosine A2b-receptor/cAMP/PKA-dependent mechanism [64,65]. Such a regulatory mechanism provides the signalling to ensure that any increase in evaporative fluid loss as either the rate or depth of breathing changes can be compensated for by increasing fluid secretion and that any increased burden of airway mucus is likewise provided with the necessary hydration. Of note, exercise and chest physiotherapy both improve clinical outcomes in CF and will also enhance calcium-mediated fluid secretory mechanisms and promote mucus clearance [63,66,67]. These data support the concept that TMEM16A can provide a meaningful level of anion and therefore fluid transport into the airways of CF patients, and although it is likely sufficient to maintain a basal level of mucus clearance, it can be overwhelmed in the absence of concomitant CFTR function. For example, during viral infections it has been demonstrated that levels of airway ATP can be reduced through accelerated metabolism resulting in attenuated TMEM16A-mediated fluid secretion with implications for mucus clearance [62].

Data support that human airways do however adapt to upregulate anion and fluid secretory capacity under disease conditions. For example, the magnitude of the NPD response elicited by purinergic agonists is reported to be greater in CF patients than in normal subjects [61]. Similarly, in vitro studies demonstrated a greater CaCC-mediated current in CF-derived HBE compared with non-CF, suggesting that the pathway may be regulated in response to disease [68,69,70]. Mechanistic data have illustrated an increase in apical ER density and a parallel increase in the magnitude of intracellular calcium response following purinergic activation of CF-HBE relative to non-CF controls, an effect that could be phenocopied by exposure of HBE to the soluble fraction of CF-derived mucopurulent material [71]. However, the effects of the mucopurulent material appear to be specific for changes in the density of ER and the magnitude of calcium signalling as TMEM16A expression is unchanged [72]. Augmented store-operated calcium entry has also been proposed as a mechanism for altered calcium handling in CF cells [73]. In contrast, the Th2 cytokines IL-4 and IL-13 and the bacterial product pyocyanin upregulate TMEM16A expression and function in the airway epithelium without influencing intracellular calcium signalling [34,74,75]. In the airway, TMEM16A expression appears to be most abundant in goblet cells, and both IL-4 and IL-13 also increase MUC5AC production [76]. Extended treatment with either Th2 cytokine can also remodel the epithelium to form a goblet cell metaplasia, a phenotype that is associated with a pronounced upregulation of CaCC function and associated fluid secretion [77]. These data are consistent with a concomitant increase in both the fluid and mucin secreting capacity of the epithelium. In further support of this, it has been demonstrated that the sodium/potassium/chloride co-transporter, NKCC1, a key element of epithelial chloride secretion, is upregulated in the basolateral membrane of airway goblet cells in the mucin hypersecreting epithelium [78]. The co-expression of TMEM16A with NKCC1 in contralateral membranes of goblet cells would be consistent with the mucin producing cell also providing anion secretion under conditions where intracellular calcium is elevated, the same signalling process that regulates goblet cell exocytosis [44].

## 4. The Discovery and Validation of TMEM16A Potentiators

**Figure 3:**
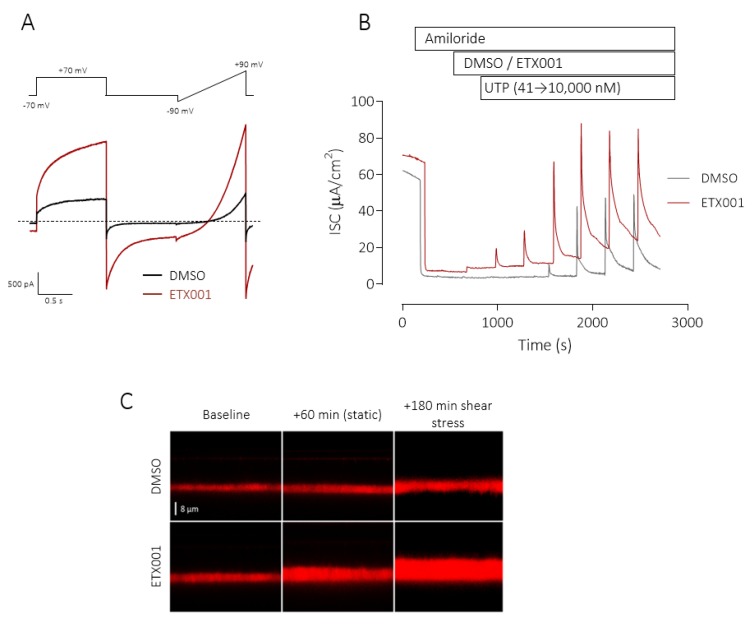
ETX001 potentiates TMEM16A function in recombinant and primary human cell systems. Whole-cell patch-clamp electrophysiological recordings from FRT cells stably expressing hTMEM16A (**A**) in the absence (black lines) and presence (red lines) of ETX001 (1.1 µM). Dotted line indicates the zero-current level. Sample raw data trace illustrating the effects of ETX001 (1 µM) upon the UTP-stimulated anion current response (**B**). After inhibition of the ENaC current with amiloride (10 µM), inserts were treated with either DMSO (grey dotted trace) or 1 µM ETX001 (red line) prior to the cumulative addition of increasing concentrations of UTP. (**C**) Sample XZ confocal images of airway surface liquid (ASL) visualised using Texas red staining in the absence and presence of ETX001 (1 µM). The change in ASL height from baseline was measured following a 1 h treatment with ETX001 or vehicle under “static conditions”, i.e., no shear stress, and also after 3 h of continual shear-stress. Reprinted from [39] with permission of the American Thoracic Society. Copyright © 2020 American Thoracic Society. The American Journal of Respiratory and Critical Care Medicine is an official journal of the American Thoracic Society.

As described above (Figure 2), the airway epithelium can acutely increase TMEM16A-mediated anion secretion and thereby fluid secretion through an increased release of ATP/UTP. Additionally, TMEM16A function can be enhanced by either increasing the calcium available for intracellular signalling or by increasing TMEM16A expression *per se*. From a therapeutic perspective, could a further potentiation of TMEM16A function provide additional hydration in the airway and associated clinical benefit? To this end, ETX001 has been recently described as a potent and selective low-molecular-weight TMEM16A potentiator [39]. Parallel hit-finding screens, including a 20,000 compound, automated patch-clamp format, identified a low-potency, low-efficacy hit that was optimised to deliver ETX001. ETX001 effectively potentiated recombinant TMEM16A in patch-clamp assays (EC_50_ = 116 nM; E_max_ ~300%) where intracellular calcium levels were tightly buffered (Figure 3A). The advantage of buffering intracellular calcium levels was that any compound-induced increase in TMEM16A-mediated current could not be due to a nonselective elevation of intracellular calcium, thereby confirming a potentiator mode-of-action. In further support of a potentiator mechanism, ETX001 and related compounds showed no activity on TMEM16A when intracellular calcium was clamped to zero. ETX001 treatment of CF-HBE enhanced short-circuit current responses to calcium-mobilising agents, including UTP and the SERCA pump inhibitor, cyclopiazonic acid (Figure 3B). As mentioned above, the good correlation between recombinant patch-clamp assay data and native TMEM16A-expressing CF-HBE further supported the identity of TMEM16A as the native airway CaCC.

Functional testing confirmed that there was no effect of ETX001 on ENaC-mediated sodium absorption or CFTR-mediated secretory currents that may have influenced downstream assays of mucosal hydration. Furthermore, as intracellular calcium levels were not buffered in the CF-HBE assays, it was important to test whether ETX001 could influence calcium signalling to correctly interpret the primary cell work. This was especially important as it has been proposed that TMEM16A plays a central role in the regulation of intracellular calcium signalling [79,80]. Using a combination of knockdown experiments as well as TMEM16A blockers, it has been reported that TMEM16A is required for GPCR-mediated calcium release from the ER and is therefore central to a host of calcium-mediated biological activities. ETX001 did not directly influence calcium levels and failed to modulate GPCR-mediated increases in intracellular calcium, further supporting a direct TMEM16A-potentiator mode-of-action in the primary cell assays and challenging the proposed role for TMEM16A in calcium regulation.

Whether the potentiation of TMEM16A function in the airways could enable a physiologically-relevant increase in fluid secretion remains a key question for the validation of both TMEM16A as a therapeutic target and also the potentiator compounds. In vitro studies have demonstrated that by imposing mechanical forces on primary cultures of CF-HBE to stimulate the endogenous release of ATP and UTP to regulate intracellular calcium levels, a substantial fluid secretory response is observed [48,62,63]. Using this system where intracellular calcium levels were physiologically regulated, ETX001 was demonstrated to further increase fluid secretion, consistent with the potentiation of TMEM16A function being sufficient to promote mucosal hydration (Figure 3C).

In vivo studies using purinergic agonists have demonstrated positive effects on mucus clearance [40,81,82], which although supportive of a role for TMEM16A in the whole animal system, are not conclusive. As described above (Figure 1), in addition to the activation of TMEM16A, the elevation of intracellular calcium stimulated by purinergic agonists will elicit additional epithelial responses that will positively influence mucus clearance [42,43,44,45]. When ETX001 was administered to sheep by inhaled dosing, the TMEM16A potentiator promoted mucus clearance both in a model with normal CFTR function as well as in a model where a CFTR inhibitor (CFTR-Inh172) was co-administered. The lack of activity of ETX001 on either ENaC or CFTR function supports the physiological relevance of TMEM16A in providing the fluid to promote airway mucus clearance in vivo.

## 5. Additional Roles for TMEM16A in the Airways: Could TMEM16A Inhibitors be a Therapeutic Approach?

Historically, activation of the airway CaCC has been considered to be of potential therapeutic benefit based on the data described above, and TMEM16A potentiator compounds such as ETX001 have continued to build the positive-modulator story. However, additional physiological roles for TMEM16A have been described, including potential functions in the airways, that may challenge the therapeutic potential of increasing TMEM16A activity [83]. These roles include the regulation of airway smooth muscle contraction and airway hyper-responsiveness, goblet cell formation and goblet cell exocytosis. TMEM16A expression has been identified in airway smooth muscle [84] and the TMEM16A blockers benzbromarone and niclosamide have been reported to attenuate human and murine airway smooth muscle contraction [52,85,86]. Benzbromarone was also demonstrated to impair mucin release from cultured human airway epithelial cells, suggesting a role for TMEM16A in the mechanism of goblet cell exocytosis [52]. A potentially related phenotype has been described in a murine *Tmem16a*^−/−^ model, where Tmem16a was silenced specifically in ciliated cells and resulted in an expansion of the goblet cell population [51]. How silencing of *Tmem16a* in a ciliated cell would induce goblet cell formation is not known, but it has been suggested that TMEM16A in murine ciliated cells may regulate the production/release of a goblet cell secretagogue, the loss of which leads to a “constipation” and apparent expansion of the secretory cell population as the trigger for secretion is lacking. In contrast, alternative literature suggests that inhibition of TMEM16A results in a reduced goblet cell population, inferring that TMEM16A channel function promotes goblet cell formation [53,54,55]. Based on these data, it has been recently suggested that inhibiting TMEM16A may present an attractive “global” approach to treating respiratory diseases associated with mucus obstruction, with an emphasis on bronchodilation and attenuated mucus production and release [83,86]. These data also infer that a positive modulator of TMEM16A function may induce the following opposite phenotypes: (1) induce bronchospasm or enhance a bronchoconstrictor response; (2) stimulate goblet cell exocytosis through either a direct effect on the secretory machinery and/or inducing the paracrine release of a mucin secretagogue; (3) promote an expansion of the goblet cell population. Compounds like ETX001 will therefore help to address these questions, and preliminary reports indicate that potentiation of TMEM16A may not induce mucin release from primary human cells or drive an expansion of the goblet cell population [87,88]. It should also be noted that many of the TMEM16A blockers in routine use are relatively impotent and nonselective compounds and that care should be taken in drawing conclusions from associated studies. For example, benzbromarone is a clinically used uricosuric drug and inhibits URAT1 function [89]. It has been demonstrated to inhibit CFTR function [90] and to be a mitochondrial toxin, reducing cellular ATP levels in the same concentration range that blocks TMEM16A (10–30 µM) [91]. Niclosamide is a hydrogen ionophore that translocates protons across the mitochondrial membrane and induces mitochondrial uncoupling [92]. Furthermore, in addition to blocking outward TMEM16A current (chloride-in), niclosamide has paradoxically been demonstrated to potentiate the inward (chloride-out) current, i.e., niclosamide appears to both potentiate and block TMEM16A [86]. So, is niclosamide blocking or potentiating TMEM16A function in the reported functional studies, or perhaps neither? Niflumic acid is a nonselective chloride channel blocker including a blocker of CFTR [93] and a nonsteroidal anti-inflammatory agent with reported activity on a range of additional ion channels [94,95,96]. Ani9, a more potent TMEM16A inhibitor (IC_50_ ~100 nM) displays selectivity within the TMEM16 family [97] and is without effect on other airway epithelial ion-transport processes; thus, it may be a more useful tool compound with which to probe TMEM16A function in future studies.

## 6. TMEM16A Function Outside of the Airways

ETX001 has been designed as an inhaled TMEM16A potentiator to maximise the opportunity for target engagement in the airway epithelium whilst minimising the systemically available dose. TMEM16A is expressed in a variety of tissues outside of the airways including vascular smooth muscle, the gastrointestinal tract (epithelium and interstitial cells of Cajal) and neuronal cells [84]. TMEM16A is also amplified and highly expressed in a number of cancers [98]. Similar to the biological function in the airways, a number of roles for TMEM16A in these tissues have been proposed based on knockout or transgenic models or through the use of nonselective TMEM16A blockers.

Calcium-activated chloride channels have been proposed to play a key role in the regulation of vascular tone by underpinning the initial depolarisation leading to the activation of voltage-operated calcium channels (VOCCs). Although these channels have been widely characterised in human and animal vascular preparations, whether TMEM16A is the vascular “CaCC” and what role it plays is far from clear [99]. However, with this caveat, studies knocking down *Tmem16a* in the vasculature [100] report changes in vascular contractility and blood pressure. Some of these effects have been recapitulated using literature-described TMEM16A blockers, although the mechanism by which this occurs has been questioned as the compounds are effective in the absence of any chloride gradient [101], consistent with a lack of selectivity of these blockers as described above. Animal studies have also implicated upregulation of *Tmem16a* as contributing to hypertension [102,103]; however, these are yet to be recapitulated in human tissues. Together, these data do suggest that upregulating TMEM16A function in vascular smooth muscle may elevate blood pressure. However, a recent report has demonstrated no effect of the overexpression of *Tmem16a* on a vascular smooth muscle cell-specific promoter [104].

Gastric emptying and gastric motility have been reported to be regulated by TMEM16A function [84,105] likely through the regulation of the pacemaker activity of the interstitial cells of Cajal. Studies have been conducted using TMEM16A blockers and knockout experiments highlighting a reduced contractility of gastric smooth muscle. Whether positive regulation of TMEM16A will increase gastric contractility is inferred but not yet tested. Knockout of *Tmem16a* in the mouse has been associated with the loss of calcium-stimulated anion secretion in the intestinal epithelium [106] and their protection from diarrhoea induced by the rotavirus enterotoxin, NSP4. The intestinal-specific knockout of *Tmem16a* resulted in accumulation of mucus in intestinal goblet cells [51]. These data may suggest a prosecretory activity of a TMEM16A potentiator in the intestines. Considering that >50% of the dose of an inhaled compound can be swallowed, these observations in the GI tract may represent important considerations for the development of inhaled TMEM16A potentiators.

The dorsal root ganglia (DRG) are another prominent site of TMEM16A expression [107]. Silencing or deletion of *Tmem16a* in DRG has been demonstrated to reduce nociceptive behaviour consistent with a salient role in sensory neurons mediating both heat [107] and inflammatory conditions [108]. TMEM16A blockers have been proposed as a novel approach to treating neuropathic pain [109], although some of the caveats associated with the selectivity of widely used TMEM16A blockers apply. A broader coverage of neuronal silencing of *Tmem16a* using a synapsin-1 targeting strategy reported impaired social behaviour, depressive traits and some weight loss. Locomotor and cognitive functions were normal, as was motor coordination [110].

Like CFTR [111], TMEM16A has been associated with several cancers and was proposed as a marker (DOG1, TAOS2) prior to its identification as a calcium-activated chloride channel [56]. The gene encoding for TMEM16A maps to chromosome 11q13, an area known to be frequently amplified in cancers. However, this amplicon also contains several well-known oncogenes, including cyclin D1 and fibroblast growth factor 19, which has complicated understanding of whether TMEM16A is an independent oncogenic factor. There is an expansive body of literature implicating TMEM16A as an oncogenic factor (reviewed in [112]) which proposes that TMEM16A can enhance proliferation and metastasis. The supporting data has been generated primarily by knockdown or overexpression studies. Pharmacological studies are scant and have used neither potent nor selective blockers. Where effective molecules have been used and thoroughly investigated, it has been suggested that inhibitory effects are mediated via internalization or degradation of the TMEM16A protein rather than through inhibition of channel function [113,114].

## 7. Summary and Concluding Remarks

Positive modulation of TMEM16A in the airways has been proposed as a mechanism to promote mucosal hydration in cystic fibrosis. Anecdotal data including the maintenance of a basal rate of mucus clearance in CF patients in addition to the clinical benefits of manoeuvres that will enhance calcium-dependent anion and fluid secretion in the lungs (exercise, chest physiotherapy, inhaled hypertonic saline) support the concept that further promoting this pathway may be a promising therapeutic approach. Preclinical data with the TMEM16A potentiator ETX001 provide evidence that the pathway can be enhanced to drive increased quantities of fluid into the airway mucosa and that in vivo there is capacity in the pathway to accelerate mucus clearance. Further studies with selective modulators of TMEM16A will be required to understand the implications of some of the other proposed biological roles of the channel in both the airways and extrapulmonary tissues. Ultimately, clinical studies with TMEM16A potentiators will define the utility of the approach for treating CF and other respiratory diseases associated with mucus obstruction.
